# Cell-permeable p38 MAP kinase promotes migration of adult neural stem/progenitor cells

**DOI:** 10.1038/srep24279

**Published:** 2016-04-12

**Authors:** Makoto Hamanoue, Kazuhito Morioka, Ikuroh Ohsawa, Keiko Ohsawa, Masaaki Kobayashi, Kayo Tsuburaya, Yoshikiyo Akasaka, Tetsuo Mikami, Toru Ogata, Ken Takamatsu

**Affiliations:** 1 Department of Physiology, Toho University School of Medicine, 143-8540, Tokyo, Japan; 2 Advanced Medical Research Center, Toho University Graduate School of Medicine, Tokyo 143-8540, Japan; 3 Brain and Spinal Injury Center (BASIC), Department of Neurological Surgery, University of California, San Francisco, 94110, California, USA; 4 Research Team for Mechanism of Aging, Redox Research, Tokyo Metropolitan Institute of Gerontology, 173–0015, Tokyo, Japan.; 5 Department of Neurochemistry, National Institute of Neuroscience, 187-8502, Tokyo, Japan; 6 Department of Pathology, Toho University School of Medicine, 143-8540, Tokyo, Japan; 7 Department of Rehabilitation for the Movement Functions, Research Institute, National Rehabilitation Center for Persons with Disabilities, 359-8555, Saitama, Japan

## Abstract

Endogenous neural stem/progenitor cells (NPCs) can migrate toward sites of injury, but the migration activity of NPCs is insufficient to regenerate damaged brain tissue. In this study, we showed that p38 MAP kinase (p38) is expressed in doublecortin-positive adult NPCs. Experiments using the p38 inhibitor SB203580 revealed that endogenous p38 participates in NPC migration. To enhance NPC migration, we generated a cell-permeable wild-type p38 protein (PTD-p38WT) in which the HIV protein transduction domain (PTD) was fused to the N-terminus of p38. Treatment with PTD-p38WT significantly promoted the random migration of adult NPCs without affecting cell survival or differentiation; this effect depended on the cell permeability and kinase activity of the fusion protein. These findings indicate that PTD-p38WT is a novel and useful tool for unraveling the roles of p38, and that this protein provides a reasonable approach for regenerating the injured brain by enhancing NPC migration.

In the embryonic brain, neural stem/progenitor cell (NPC) migration is necessary for normal brain development^1^, and a lack of NPC migration causes severe brain damage and lethality^2^,^3^. In the adult brain, NPCs are localized to the subventricular zone (SVZ) and the subgranular zone (SGZ) of the hippocampus, from which they migrate to the olfactory bulb through the rostral migratory stream (RMS) and the granular cell layer of the dentate gyrus of the hippocampus, respectively^4^. On the other hand, in the acutely injured brain, adult NPCs from the SVZ migrate to sites of injury through blood vessels or neuronal fibers for up to 1 year after injury^5^,^6^,^7^; however, the number of migrated cells is low relative to the number of residual cells in the injured site (max. 2%)^6^. These observations indicate that NPC migration after injury is an endogenous regeneration response, and suggest that enhancement of this NPC migration could be useful for regeneration of damaged brain.

Many factors promote NPC migration to sites of injury: stromal cell–derived factor (SDF-1)^8^,^9^,^10^,^11^, hepatocyte growth factor^12^, insulin-like growth factor-1^13^, stem cell factor^14^, monocyte chemotactic protein-1^14^, and vascular endothelial growth factor^15^. These extracellular factors converge on several intracellular signaling factors, some of which are considered to be intracellular candidates for enhancers of NPC migration: cyclin-dependent kinase 5 (Cdk5)^3^,^16^, doublecortin (Dcx)^17^, c-Jun NH_2_-terminal kinase (JNK)^18^, extracellular signal-regulated kinases 1 and 2 (ERK1/2)^19^, protein kinase C^20^, RhoA^21^, RhoC^22^, and Wnt/β-catenin^10^. Among these factors, ERK1/2 and JNK belongs to mitogen-activated protein (MAP) kinase family, and their expression is induced after injury of brain neurons^23^. p38 MAP kinase (p38, also known as stress-activated protein kinase 2 [SAPK2]), is another component of the MAP kinase family, and its expression is also elevated after injury of neurons^24^, astrocytes^24^, and microglia^25^ in the brain. Chemical inhibition experiments demonstrated that sustained activation of p38 is associated with neuronal death and apoptosis^26^,^27^. In NPCs, expression of p38 is detectable from mouse embryonic day 10^28^, and may play regulatory roles in NPC proliferation^28^,^29^,^30^,^31^, apoptosis^32^,^33^, chemokine production^34^, and cell survival^35^. p38 participates in migration of several types of cells, including cortical neurons^36^, HeLa cells^37^, and mesoderm^38^; however, the role of p38 in NPC migration remains incompletely understood.

In this study, chemical inhibition experiments revealed that endogenous p38 supports cell migration of cultured NPCs obtained from adult brain. Furthermore, a cell-permeable wild-type p38 protein promoted random cell migration of adult NPCs. These results suggest that direct introduction of p38 into adult NPCs, resulting in enhanced NPC migration to sites of injury, represents an alternative approach for regenerating damaged brain.

## Results

### p38 MAP kinase expression in adult brain and cultured adult NPCs

**Figure 1:**
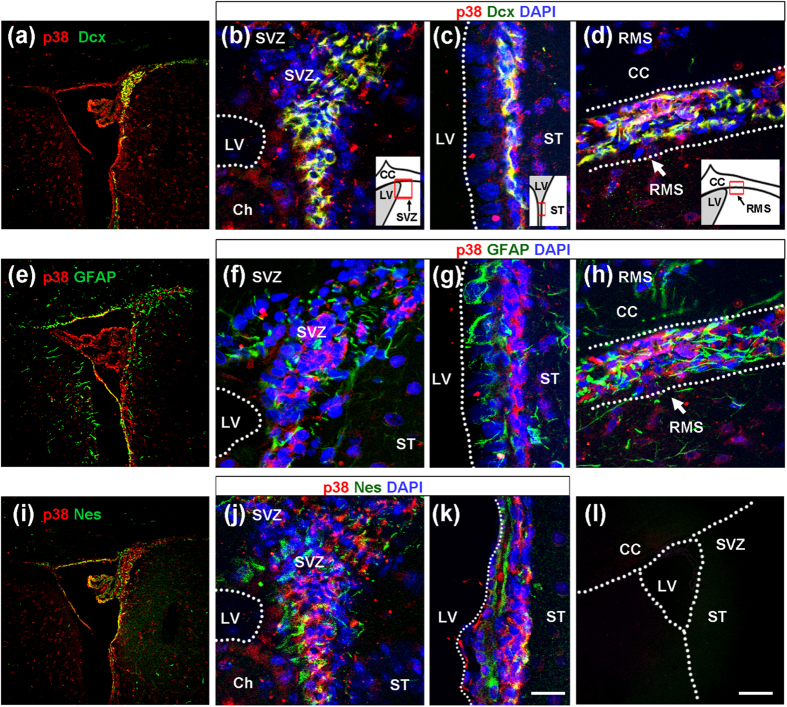
p38 protein is expressed in doublecortin (Dcx)-positive NPCs in the adult brain. Brain slices were prepared from adult mice, and immunohistochemical analysis was performed with anti-p38, anti-Dcx, anti-GFAP, and anti-nestin antibodies. p38 was expressed specifically in Dcx-positive NPCs of the subventricular zone (**a–c**) and RMS (**d**) rather than in GFAP- (**e–h**) or nestin-positive (**i–k**) neural stem cells. (**l**) Negative control staining using only secondary antibody. CC, corpus callosum; LV, lateral ventricle; ST, striatum; SVZ, subventricular zone. Scale bar = 20 μm (**b–k**), 100 μm (**a,e,i,l**).

Previously, we showed that p38 is predominantly expressed in NPCs obtained from mouse brain at embryonic day 10, and that its expression decreases gradually during development^28^. To determine whether NPCs express p38 in adult brain, we performed immunohistochemical analysis with an anti-p38 antibody (Fig. 1). p38 expression was observed in ventricular cells, subventricular cells, and choroid plexus cells in the adult brain (Fig. 1a). Especially in SVZ and RMS, most p38-positive cells expressed doublecortin, a marker of migrating progenitor cells^39^ (Fig. 1a–d). In these areas, p38-positive cells expressed no or very low levels of the neural stem cell / astrocyte antigens GFAP (Fig. 1e–h) and nestin (Fig. 1j–k). These results suggest that p38 plays regulatory roles in NPC migration.

**Figure 2:**
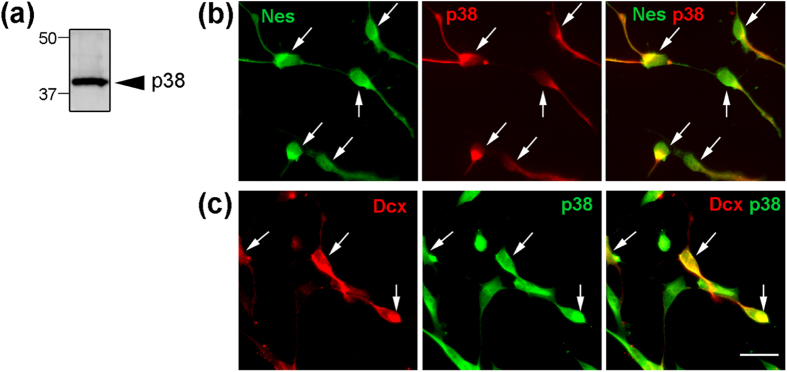
p38 is expressed in cultured NPCs obtained from adult brain. (**a**) Western blot analysis of proteins (10 μg) from adult NPCs, using an anti-p38 antibody, revealed expression of p38 in cultured NPCs. (**b,c**) Immunocytochemical analysis of dissociated NPCs was performed using anti-nestin and anti-p38 or anti-Dcx antibodies. Adult cultured NPCs expressed nestin (**b**), and some also expressed doublecortin (**c**). Both nestin- and Dcx-positive NPCs expressed p38 (**b,c**, arrows). Scale bar = 20 μm.

To determine the effect of p38 on NPC migration, we prepared cultured NPCs from postnatal (8-week old) adult brain. The essential phenotypes of NPCs, i.e., their self-renewal capacity and multipotency, were verified in the previous reports^40^,^41^. Western blot analysis revealed that p38 was expressed in cultured adult NPCs (Fig. 2a). Immunocytochemical analysis revealed that our NPCs expressed nestin antigen, a specific marker of NPCs (Fig. 2b, Supplemental Fig. 1a, 89.8 ± 6.6%, n = 116). Some of these NPCs also expressed doublecortin, which is specific for migrating NPCs (Fig. 2c, Supplemental Fig. 1b, 31.5 ± 1.7%, n = 132). Almost no cells expressed Mash-1 or NeuN, both of which are specific for intermediate progenitors and neurons, respectively (data not shown). Both nestin-positive NPCs and Dcx-positive NPCs expressed p38 (Fig. 2b,c) and phosphorylated p38, the activated form of p38 (Supplemental Fig. 1a,b). These results suggest that in adult NPCs, expression of p38 plays a regulatory role in processes such as proliferation, differentiation, and migration.

### Spontaneous migration activity of endogenous p38 MAP kinase

**Figure 3:**
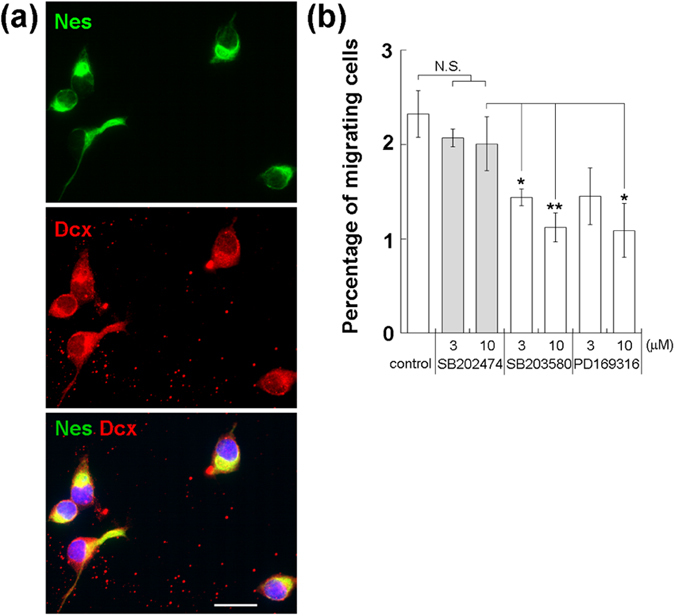
Endogenous p38 induces spontaneous migration of adult NPCs. (**a**) NPCs that migrated from the insert to the coverslip in the receiver plate of Transwell were stained with anti-nestin and anti-Dcx antibodies. Migrated NPCs expressed nestin and Dcx. Scale bar = 20 μm. (**b**) Adult NPCs were seeded in Transwell inserts and cultured in growth-promoting medium containing the specific p38 inhibitors SB203580 and PD169316, and the number of migrated NPCs in the receiver plate was determined by measuring intracellular ATP. Data are means ± SEM from four independent experiments. SB203580 and PD169316 inhibited adult NPC migration relative to that in control cells and cells treated with SB202474, an inactive analog of SB203580. *P < 0.05, **P < 0.01, N.S.: not significant.

To assess the function of p38, which is expressed endogenously in adult NPCs, we conducted cell migration assays using Transwell apparatus. To identify the cells that migrated from the insert to the receiver plate of the Transwell, we removed the coverslips from the receiver plate 16 hr after the cells were seeded, and then subjected them to immunocytochemistry (Fig. 3a). Almost all migrated cells expressed both nestin (99.5 ± 0.6%, n = 122) and doublecortin (Dcx, 99.4 ± 0.7%), suggesting that nestin- and doublecortin-positive adult NPCs could migrate from the insert to the receiver plate of the Transwell apparatus. To quantitate the migration activity of adult NPCs, cells in the receiver plate and the insert were counted 16 hr after seeding by measurement of intracellular ATP. Relatively few of the seeded cells migrated to the receiver plate (2.2 ± 0.2%, n = 8). The p38-specific inhibitors SB203580 and PD169316 significantly reduced the number of migrating adult NPCs relative to those in control cells or cells treated with SB202474, an inactive analog of SB203580 (Fig. 3b, n = 4). These results suggest that endogenous p38 could participate in spontaneous migration of adult NPCs.

### Characterization of a cell-permeable p38 MAP kinase protein

**Figure 4:**
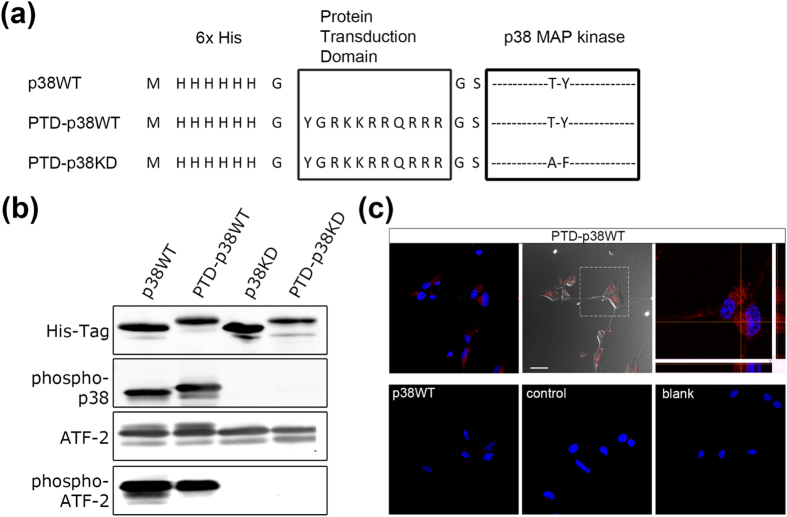
Characterization of cell-permeable p38 MAP kinase protein. (**a**) Construction of cell-permeable p38 proteins. PTD-p38WT contains the PTD (protein transduction domain) and p38WT (wild-type p38). p38KD is a kinase-dead form of p38. (**b**) *In vitro* kinase assay revealed that both PTD-p38WT and p38WT were activated by autophosphorylation (phospho-p38), and that both proteins phosphorylated ATF-2, a known substrate of p38 (phospho-ATF-2). p38KD and PTD-p38KD did not exhibit kinase activity. (**c**) Cultured adult NPCs were treated with p38 proteins (600 nM, 24 hr), and immunocytochemical staining was performed with anti-His-tag antibody. Positive staining was detected in NPCs treated with PTD-p38WT (upper lane), but not in cells treated with p38WT, cells not treated with proteins (control), or in cells stained only with secondary antibody (blank). Scale bar = 20 μm.

In light of the observation that NPC migration was blocked by a p38 inhibitor, we hypothesized that increasing the intracellular concentration of p38 protein might promote adult NPC migration. In order to introduce p38 protein into adult NPCs, we generated a cell-permeable p38 protein by fusing the HIV protein transduction domain (PTD), an 11–amino acid sequence^42^, to the N-terminus of p38 (Fig. 4a). Several versions of this construct were prepared: cell-permeable wild-type p38 (PTD-p38WT), in which the dual phosphorylation site (Thr-180/Tyr-182) required for kinase activity was preserved; cell-permeable kinase-dead p38 (PTD-p38KD), in which Thr-180 and Tyr-182 were mutated to alanine (A) and phenylalanine (F), respectively; and cell-impermeable wild-type p38 (p38WT). These recombinant p38 proteins were purified from *E. coli* as histidine-tagged proteins, and blotted with anti-phospho-p38 antibody to verify their activation status. Western blot analysis revealed that purified p38WT and PTD-p38WT were activated by autophosphorylation (Fig. 4b, phospho-p38), and that both proteins were able to phosphorylate ATF-2 peptide, a substrate of p38 (Fig. 4b, phospho-ATF-2). By contrast, p38KD and PTD-p38KD, which were kinase-dead and not autophosphorylated, could not phosphorylate ATF-2 peptide. To verify the cell permeability of PTD-p38WT, we treated cultured adult NPCs with this protein for 24 hr, and then stained them with anti-His-tag antibody. Confocal microscopy revealed positive intracellular staining for the His-tag following treatment with PTD-p38WT. No staining was observed following treatment with p38WT, which lacks the cell-permeable PTD domain, or in control cells (Fig. 4c). These results indicated that recombinant PTD-p38WT has constitutive kinase activity and can penetrate NPCs.

### Cell-permeable p38 protein promotes random migration of adult NPCs

**Figure 5:**
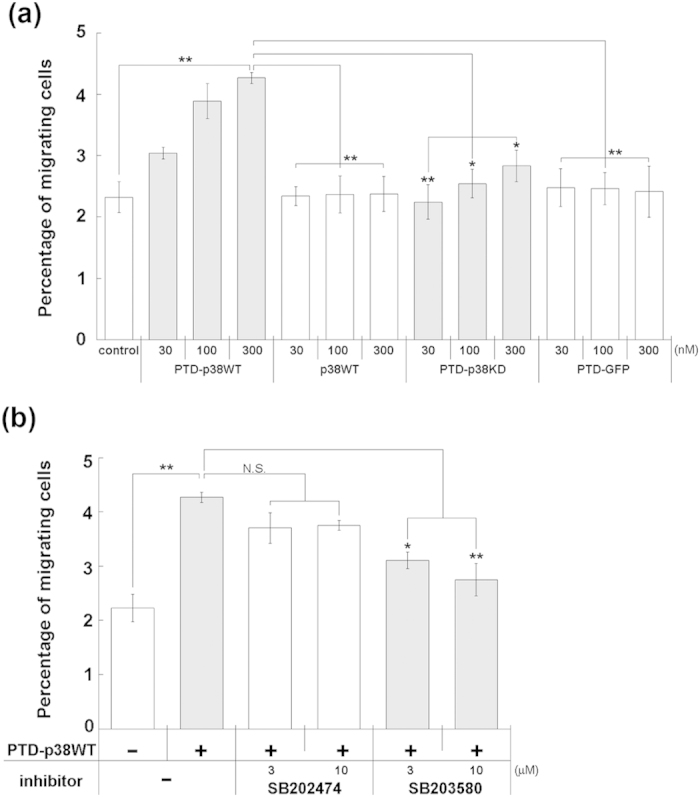
Cell-permeable p38 protein enhances adult cultured NPC migration. The number of NPCs that migrated to the receiver plate from the Transwell insert was measured by intracellular ATP. Migration ratio of adult NPCs was calculated by dividing the number of migrated cells by the total cell number. Data are means ± SEM. (**a**) PTD-p38WT significantly increased adult NPC migration relative to that in control cells (n = 7) or cells treated with p38WT, PTD-p38KD, or PTD-GFP (n = 4). (**b**) Activity of PTD-p38WT was blocked by the specific p38 inhibitor SB203580. *P < 0.05, **P < 0.01, N.S.: not significant.

To determine whether direct introduction of cell-permeable p38 protein would enhance NPC migration, we conducted Transwell assays. Specifically, cultured adult NPCs were seeded into Transwell inserts and maintained for 16 hr in the presence of recombinant proteins. The migration ratio of adult NPCs treated with PTD-p38WT was calculated by dividing the number of migrated cells by the total cell number. PTD-p38WT significantly increased the migration ratio relative to that of control cells (Fig. 5a, 300 nM, n = 7), but the ratio was unchanged in NPCs treated with p38WT, which is not cell-permeable. Likewise, neither PTD-p38KD, which lacks kinase activity, nor PTD-GFP (consisting of the His-Tag, PTD-domain, and GFP) changed the migration ratio. Furthermore, the p38-specific inhibitor SB203580 blocked the migration-enhancing activity of PTD-p38WT, whereas its inactive analog SB202474 did not (Fig. 5b). These results suggest that both the cell permeability and kinase activity of PTD-p38WT were necessary for enhancement of adult NPC migration.

Next, to determine whether the increase in the number of cells in the receiver after 16 hr cultivation was a consequence of changes in NPC survival, we measured the numbers of living cells in both the Transwell insert and the receiver plate, and then calculated the ratio between the number of living cells in the presence of PTD-p38WT and the number in control samples. Treatment with recombinant proteins did not cause changes in cell number after 16 hr (Supplemental Fig. 2a) or after 5 days of plating (Supplemental Fig. 2b). These results indicate that direct introduction of p38 activity by treating cells with PTD-p38WT promotes adult NPC migration without affecting NPC survival.

**Figure 6:**
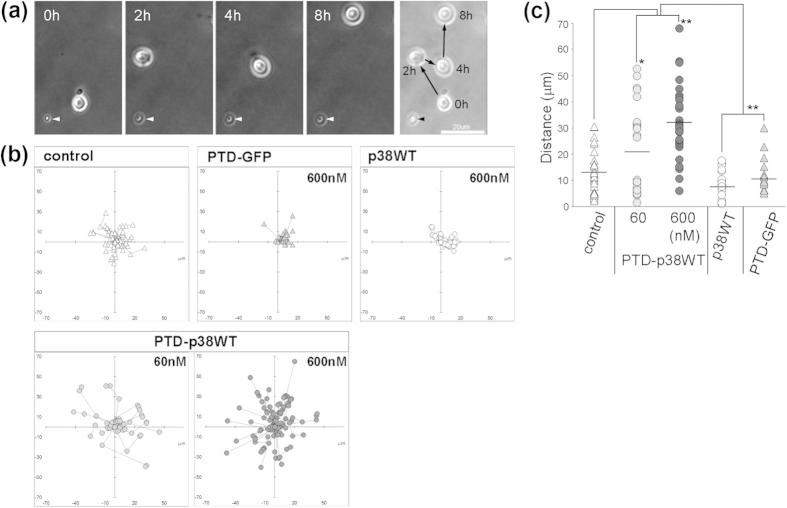
Cell-permeable p38 protein promotes random migration of adult cultured NPCs. (**a**) Time-lapse video microscopy shows migration of adult NPCs within 8 hr. Arrow in the right photograph shows the migration route. The arrowhead in every photograph is cell debris, used as a fiducial marker for cell migration. Scale bar = 20 μm. (**b**) Migrational trajectories of adult NPCs. Adult NPCs treated with PTD-p38WT migrated more randomly than control cells or cells treated with p38WT or PTD-GFP. (**c**) PTD-p38WT increased the number of migrated cells and the migration distance relative to that in control cells or cells treated with p38WT or PTD-GFP. *P < 0.05, **P < 0.01.

To investigate the migration-promoting activity of PTD-p38WT in detail, we performed a time-lapse analysis of adult NPCs using a Dunn chamber. In cells treated with PTD-p38WT, migration of adult NPCs was frequently observed over 8 hr (Fig. 6a). Adult NPCs treated with PTD-p38WT migrated for greater distances than cells treated with control or p38WT, but their migration was random rather than in unidirectional (Fig. 6b). PTD-p38WT increased the number of migrated cells relative to that in control cells or cells treated with p38WT or PTD-GFP, and the distance traveled by adult NPCs treated with PTD-p38WT was 2.7-fold higher than that of controls (Fig. 6c; 32.3 ± 2.5 μm, n = 31 vs. 11.9 ± 1.5 μm, n = 32, respectively). These results suggest that PTD-p38WT potently promotes random rather than chemotactic migration.

To determine whether cell-permeable p38 protein influenced adult NPC differentiation, we monitored differentiation status after a 5-day treatment, using Western blot analysis to detect cell type–specific antigens^40^: brain lipid binding protein (BLBP) for NPCs, Dcx for migrating NPCs, NeuN for neurons, and GFAP for astrocytes (Supplemental Fig. 3). Protein levels were normalized against actin. No significant changes in expression levels of these cell-specific antigens were observed between cells treated with PTD-p38WT and control cells (Supplemental Fig. 3a–h). Only PTD-p38KD treatment reduced GFAP expression (Supplemental Fig. 3g,h). Together, these results demonstrate that PTD-p38WT does not disrupt adult NPC differentiation.

## Discussion

The p38 MAP kinase family consists of four splice variants (α, β, γ, δ^43^). Of these, we investigated the function of p38α, because its expression is observed in SVZ-derived NPCs from embryonic day 10 to postnatal brain^28^. Our immunochemical experiments showed that p38α is locally expressed in doublecortin-positive adult NPCs, especially at the SVZ, and specific inhibition of p38 revealed that endogenous p38 participates in spontaneous migration of adult NPCs. The involvement of p38 protein in NPC migration prompted us to evaluate whether elevated levels of p38 could enhance NPC migration. To increase the intracellular concentration of p38, we used a cell-penetrating peptide to efficiently deliver the protein to cells (see review^44^). PTD-p38WT was able to significantly and expeditiously enhance migration of adult NPCs, whereas neither p38WT nor PTD-p38KD had this effect. Furthermore, the migration-enhancing activity of PTD-p38WT was blocked by specific inhibition of p38. These results indicate that both kinase activity and cell permeability are necessary for enhancement of NPC migration. Taken together with our findings that PTD-p38WT did not disturb cell survival or differentiation of adult NPCs, these observations show that this protein provides a safe and effective method for regenerating the injured brain by exploiting an endogenous regeneration response.

We also demonstrated that cell-permeable p38 protein enhances random migration, rather than chemotactic migration. Chemotactic migration, also known as chemotaxis, is a fundamental cell process characterized by both the speed and direction of migration^45^. SDF-1, a well-known inflammatory chemoattractant for NPCs, both increases migration speed and defines the direction of movement^8^. In addition, SDF-1 induces phosphorylation of p38 in NPCs^8^. Recently, Chen *et al.* showed that a specific inhibitor of p38 decreased the speed of migration, but not its directionality, in C17.2, a clonally multipotent neural precursor cell line derived from mouse cerebellum^46^. This observation supports our findings that p38 contributes to the magnitude of migration, but does not contribute to chemotaxis. Because intrinsic SDF-1 also promotes NPC homing after brain injury^8^,^9^, a combination of PTD-p38WT with SDF-1 and/or growth factors^47^ could strongly enhance NPC homing to sites of injury.

In this report, we showed that PTD-p38WT effectively enhances NPC migration without affecting cell survival or differentiation. However, many previous studies showed that p38 protein inhibits NPC proliferation^28^,^29^,^30^,^31^ and facilitates NPC differentiation into astrocytes^48^. These studies used p38-specific inhibitors such as SB203580 and SB202190, and/or forced expression systems involving expression plasmids or shRNA constructs. A plausible explanation for this discrepancy is that the intracellular concentration of p38 protein differs between our system (using a cell-permeable protein) and forced expression systems. Because cell-permeable proteins enter cells by inefficient mechanisms such as direct translocation and specific forms of endocytosis, and their intracellular levels are consequently lower than those resulting from forced exogenous expression^42^, the intracellular concentration of cell-permeable p38 might not be high enough to influence cell proliferation or differentiation. The low incorporation of the cell-permeable proteins might also account for the small percentage of migrating NPCs (Fig. 5a, about 4% at 300 nM PTD-p38WT) and for the lack of a dominant-negative activity of the kinase-dead form of cell-permeable p38 (Fig. 5a, PTD-p38KD).

Another plausible explanation involves the low specificity of p38 specific inhibitors: because these “specific” inhibitors block many targets other than p38 at concentrations above 5 μM^49^, care must be taken to interpret the effects of these inhibitors on p38 function in NPCs. In light of our result demonstrating that PTD-p38KD reduced GFAP expression (Supplemental Fig. 3g,h), which is supported by the results of a previous study using a specific p38 inhibitor^48^, it will be necessary to evaluate p38 functions, including effects on proliferation and differentiation *in vivo*, using cell-permeable p38. Collectively, our results also suggest that cell-permeable p38 proteins represent a novel, useful tool for unraveling the roles of p38.

Cell-permeable p38 proteins, however, have a major drawback: low cell selectivity^44^. p38 plays various roles in brain function: neurite formation^50^, induction of neuronal apoptosis^51^; production of IL-6^52^ or TNF-α^53^ from astrocytes; survival of oligodendrocytes^49^; production of GDNF^54^, NO^55^, TNF-α^53^, IL-10^56^ from microglia; and phagocytosis of microglia^57^. In particular, allodynia, which is induced by elevation of intracellular p38 in microglia^58^,^59^, is a serious consideration in the context of therapeutic compounds. Future studies are needed to determine whether low-dose PTD-p38WT can influence the function of neurons and glial cells *in vitro* and *in vivo*. Furthermore, improvements in cell permeability, such as insertion of homing sequences into the PTD domain or replacement of the PTD with an activatable cell-permeable domain^44^, may be necessary for future therapeutic applications.

In summary, we found that endogenous p38 is an intrinsic factor that promotes adult NPC migration. Furthermore, we showed that PTD-p38WT can promote migration by adult NPCs. Our findings suggest that PTD-p38WT is a novel, useful tool for unraveling the roles of p38, and that this protein could be used to regenerate damaged brain by enhancing NPC migration.

## Methods

### Ethics Statement

All animal-based experiments were performed according to the Guiding Principles for the Care and Use of Animals approved by the Council of the Physiological Society of Japan. The Ethics Review Committee for Animal Experimentation at Toho University also approved all experimental protocols used in this study (No. 14-53-189).

### NPC preparation

Adult mice (C57BL/6J) were purchased from Sankyo Laboratory Service (Tokyo, Japan). Adult NPCs were prepared from adult (8-week-old) mouse brains as described previously^40^,^41^ with slight modifications. In brief, adult brains (cerebral hemispheres and striatum) were mechanically dissociated by trituration into single-cell suspensions in Hanks’ Balanced Salt Solution (HBSS) without Ca^2+^ or Mg^2+^. Dissociated brain cells were cultured for 7 days in growth-promoting medium consisting of Dulbecco’s Modified Eagle’s Medium: Nutrient Mixture F-12 Ham (DMEM/F-12 medium), B27 supplement (Invitrogen, Carlsbad, CA, USA), epidermal growth factor (EGF; 20 ng/mL; PeproTech EC, London, UK), basic fibroblast growth factor (FGF-basic; 20 ng/mL, PeproTech EC), and penicillin and streptomycin (100 U/mL and 100 μg/mL, respectively). Cultured NPCs were obtained by dissociation of secondary neurospheres^40^,^41^.

NPC phenotype, capacity for self-renewal, and multipotency were verified by neurosphere assay^41^ and Western blot analysis^40^. All culture materials were purchased from Nalge Nunc International (Rochester, NY, USA) unless otherwise noted. All chemical reagents were purchased from Sigma-Aldrich (St. Louis, MO, USA) and Wako Pure Chemicals Industries (Osaka, Japan) unless otherwise noted. All culture materials were purchased from Greiner Bio-One (Tokyo, Japan) unless otherwise noted.

### Histochemical analysis

Immunohistochemistry was performed as described previously^60^ with slight modifications. In brief, 4-μm adult brain sections were prepared from paraffin-embedded tissues. The slices were blocked with blocking buffer (Blocking One Histo; Nacalai Tesque, Kyoto, Japan) for 20 min at room temperature following antigen retrieval (Antigen Retrieval Solution, pH9; Nichirei Biosciences Inc., Tokyo, Japan) and permeabilization (phosphate-buffered saline containing 1% Triton X-100). Sections were then incubated for 24 hr at 4 °C with anti-p38α (sc-535, rabbit polyclonal; Santa Cruz Biotechnology, Dallas, TX, USA), anti-doublecortin (sc-8066, goat polyclonal; Santa Cruz Biotechnology), anti-GFAP (glial fibrillary acidic protein, G3893, mouse monoclonal IgG; Sigma-Aldrich), and anti-nestin (2Q178, mouse monoclonal IgG; Abcam, Cambridge, UK) antibodies. After washing, the sections were incubated in buffer with biotin-conjugated anti-goat antibody (Vector Laboratories, Burlingame, CA, USA), DyLight 649–conjugated Streptavidin (Molecular Probes Life Technologies, Thermo Fisher Scientific, Waltham, MA, USA) or Alexa Fluor 488 and 555–conjugated secondary antibodies (Molecular Probes, Life Technologies, Thermo Fisher Scientific). The nuclei were visualized with 13 ng/mL of 4′,6-diamidino-2-phenylindole (DAPI). Immunofluorescence images were collected with a laser scanning microscope (A1^+^; Nikon, Tokyo, Japan).

### Immunocytochemical analysis

Immunohistochemistry was performed as described previously^28^ with slight modifications. Cultured NPCs on poly-L-lysine/ laminin-coated coverslip were fixed in 4% PFA for 10 min at room temperature, and then blocked with blocking buffer containing 1.5% normal goat serum and 0.5% Triton X-100 in PBS. Cells were stained for 24 hr at 4 °C with the following antibodies: anti-doublecortin, anti-nestin (MAB353, mouse monoclonal IgG1, Millipore, CA, USA), anti-p38α, anti-phospho-p38 MAPK (#4631, rabbit monoclonal IgG, Cell Signaling Technology, TX, USA). After washing, the cells were then stained with secondary antibodies conjugated with Alexa Fluor 488, Alexa Fluor 555, and Cy3 (all from Molecular Probes, Life Technologies, Thermo Fisher Scientific) for 1 hr at room temperature. Fluorescence images were collected using a laser scanning microscope (LSM510 META; Carl Zeiss, Oberkochen, Germany and A1^+^; Nikon). Stained cells were counted in five randomly selected fields.

To demonstrate incorporation of cell-permeable proteins, cells were treated with medium containing proteins (600 nM) for 24 hr at 37 °C. Fixed cells were blocked with Blocking One Histo (Nacalai Tesque, Fig. 4c), incubated for 6 hr at 4 °C with anti-His-Tag antibody (#12698, rabbit monoclonal; Cell Signaling Technology), and then incubated for 1 hr at room temperature with Alexa Fluor 555–conjugated secondary antibody (Molecular Probes, Life Technologies, Thermo Fisher Scientific). Fluorescence, differential interference contrast, and Z-section images were collected using a laser scanning microscope (A1^+^; Nikon).

### Western blot analysis

After washing cells with PBS, protein samples were prepared from cells with lysis buffer containing 10 mM Tris-HCl [pH 7.5], 150 mM NaCl, 1 mM EDTA, 1% NP40, 1 mM Na_3_VO_4_, and Halt protease inhibitor cocktail (Thermo Fisher Scientific). The protein samples were boiled with Laemmli’s sample buffer, separated on 5–15% Next Page (Gellex International, Tokyo, Japan), and transferred to polyvinylidene difluoride (PVDF) membranes, which were then incubated in Blocking One-P (Nacalai Tesque). After washing, the membranes were incubated for 16 hr at 4 °C with the following antibodies: anti-p38α, anti-His-Tag (#2366, mouse monoclonal IgG1, Cell Signaling Technology), anti-phospho-p38 MAPK (Thr-180/ Tyr-182) (#9211, rabbit polyclonal IgG, Cell Signaling Technology), anti-phospho-ATF-2 (#9221, rabbit polyclonal, Cell Signaling Technology), anti-ATF-2 (#9222, rabbit polyclonal, Cell Signaling Technology), anti-BLBP (ABN-14, rabbit polyclonal, Millipore), anti-NeuN/FOX3 (M-377-100, mouse monoclonal IgG, Biosensis Pty, South Australia, Australia), anti-doublecortin (611706, mouse IgG, BD Transduction Laboratories, Becton, Dickinson Company, Franklin Lakes, NJ, USA), anti-GFAP, or anti-β-actin (sc-47778, mouse monoclonal IgG1, Santa Cruz Biotechnology). Next, the blots were incubated with peroxidase-conjugated AffiniPure F(ab′)_2_ fragment goat anti-mouse IgG (115-036-072, Jackson ImmunoResearch Laboratories, West Grove, PA, USA) and peroxidase-conjugated AffiniPure F(ab′)_2_ fragment goat anti-rabbit IgG (115-036-047, Jackson ImmunoResearch Laboratories). Chemiluminescence signals were developed by the Immunostar LD kit (Wako Pure Chemicals), and were visualized by the ImageCapture G3 system (Liponics, Tokyo, Japan). Relative intensities were obtained by densitometry using a computerized analysis system (NIH ImageJ).

### Construction and preparation of PTD-p38WT

The expression plasmid pTAT-HT was constructed by inserting oligomeric nucleotides encoding the 6xHis tag and the 11–amino acid Tat-sequence (YGRKKRRQRRR)^61^ flanked by glycine residues into vector pET-3d (Novagen, Merck KGaA, Madison, WI, USA). pTAT-p38WT vectors were constructed by inserting a PCR fragment encoding the wild-type p38 open reading frame^62^ into the *Bam*HI site of the pTAT-HT vector. The pTAT-p38KD vectors were constructed by inserting a PCR fragment encoding the open reading frame of a mutant p38 protein in which the dual phosphorylation sites required for kinase activity, threonine-180 and tyrosine-182, were replaced with alanine and phenylalanine, respectively^63^. The plasmid construct was confirmed by DNA sequencing. PTD fusion proteins were expressed in Single Step (KRX) competent cells (Promega, Madison, WI, USA). PTD fusion proteins were recovered by sonication of KRX cells (250 ml) in 25 ml of equilibration/wash buffer (50 mM sodium phosphate [pH 8.0], 300 mM NaCl), and the supernatant was loaded onto TALON Metal Affinity Resins (Clontech Laboratories, Mountain View, CA, USA). The PTD fusion proteins were eluted from the resins using imidazole (0.5 M) under native conditions, and then purified using Vivaspin (5000 MWCO; Sartorius AG, Goettingen, Germany) and Zeba Spin desalting columns (Thermo Fisher Scientific).

### *In vitro* kinase assay

Kinase reactions were performed by mixing PTD fusion proteins (250 ng) with recombinant ATF-2 protein (500 ng) in the reaction solution (200 μM ATP, 25 mM Tris buffer [pH 7.5], 5 mM β-glycerolphosphate, 2 mM DTT, 0.1 mM Na_3_VO_4_, 10 mM MgCl_2_) at 30 °C for 30 min. Kinase activity was verified by Western blot analysis using anti-phospho-ATF-2 and anti-ATF-2 antibodies after the reaction solution was boiled.

### Cell viability assay

The number of viable cells was assessed by measuring intracellular ATP to identify metabolically active cells, using the CellTiter-Glo luminescent cell viability assay (Promega). The number of viable cells after 5 days was assessed by measuring intracellular WST-8 formazan using the Cell Counting Kit-8 (Dojindo Laboratories, Kumamoto, Japan).

### Transwell assay

Cell migration capacity was investigated using Transwell permeable supports with 3.0-μm pore polyester membrane inserts (No. 3472, Corning Life Sciences, Tewksbury, MA, USA) according to the manufacturer’s instructions with slight modifications. In brief, the cultured NPCs (4 × 10^3 ^cells per insert) were seeded in Transwell inserts and incubated in growth-promoting medium containing PTD proteins for 16 hr. The number of non-migrating cells (i.e., cells remaining within the inserts) or the number of migrating cells (i.e., the cells on the receiver plate) was determined using the CellTiter-Glo luminescent cell viability assay. The migration ratio of adult NPCs was calculated by dividing the number of migrated cells by the total cell number. For immunocytochemical assay of migrated cells, a coated coverslip was inserted into the receiver plate before the cells were plated.

### Dunn chemotaxis assay

To investigate migration speed and movement direction of NPC, the Dunn Chemotaxis Chamber (Weber Scientific International, Teddington, UK) assay was performed as previously described^64^. In brief, cultured NPCs were seeded at a density of 40 cells per mm^2^ on the glass coverslip coated with 1 μg/ml poly-D-lysine and 10 μg/ml laminin for 16 hr in the growth-promoting medium. The coverslip was inverted onto the Dunn chamber, and the outer and inner wells were filled with growth-promoting medium containing recombinant proteins at the indicated concentrations. Cell migration was assessed by time-lapse video microscopy over an 8 hr period (IX81, Olympus, Tokyo, Japan). The migration distance of individual cells was measured using the MetaMorph software (ver. 7.5.2.0, Molecular Devices, Sunnyvale, CA, USA). Dividing cells were excluded from the analysis.

### Statistical analysis

Statistical evaluations were performed using Student’s t-test. P < 0.05 was considered to indicate a statistically significant difference.

## Additional Information

**How to cite this article**: Hamanoue, M. *et al.* Cell-permeable p38 MAP kinase promotes migration of adult neural stem/progenitor cells. *Sci. Rep.* **6**, 24279; doi: 10.1038/srep24279 (2016).

## Supplementary Material

Supplementary Information

## References

[b1] Huang Z. Molecular regulation of neuronal migration during neocortical development. Mol. Cell. Neurosci. 42, 11–22, 10.1016/j.mcn.2009.06.003 (2009). 19523518

[b2] Gleeson J. G. *et al.* Doublecortin, a brain-specific gene mutated in human X-linked lissencephaly and double cortex syndrome, encodes a putative signaling protein. Cell 92, 63–72, S0092-8674(00)80899-5 (1998). 9489700 10.1016/s0092-8674(00)80899-5

[b3] Ohshima T. *et al.* Migration defects of cdk5(−/−) neurons in the developing cerebellum is cell autonomous. J. Neurosci. 19, 6017–6026 (1999). 10407039 10.1523/JNEUROSCI.19-14-06017.1999 PMC6783065

[b4] Cayre M., Canoll P. & Goldman J. E. Cell migration in the normal and pathological postnatal mammalian brain. Prog. Neurobiol. 88, 41–63, 10.1016/j.pneurobio.2009.02.001 (2009). 19428961 PMC2728466

[b5] Kojima T. *et al.* Subventricular zone-derived neural progenitor cells migrate along a blood vessel scaffold toward the post-stroke striatum. Stem Cells 28, 545–554, 10.1002/stem.306 (2010). 20073084

[b6] Kreuzberg M. *et al.* Increased subventricular zone-derived cortical neurogenesis after ischemic lesion. Exp. Neurol. 226, 90–99, 10.1016/j.expneurol.2010.08.006 (2010). 20713052

[b7] Osman A. M., Porritt M. J., Nilsson M. & Kuhn H. G. Long-term stimulation of neural progenitor cell migration after cortical ischemia in mice. Stroke 42, 3559–3565, 10.1161/STROKEAHA.111.627802 (2011). 21980195

[b8] Imitola J. *et al.* Directed migration of neural stem cells to sites of CNS injury by the stromal cell-derived factor 1alpha/CXC chemokine receptor 4 pathway. Proc. Natl. Acad. Sci. USA 101, 18117–18122, 10.1073/pnas.0408258102 (2004). 15608062 PMC536055

[b9] Lim T. C. *et al.* Chemotactic recruitment of adult neural progenitor cells into multifunctional hydrogels providing sustained SDF-1alpha release and compatible structural support. FASEB J. 27, 1023–1033, 10.1096/fj.12-221515 (2013). 23193174

[b10] Luo Y., Cai J., Xue H., Mattson M. P. & Rao M. S. SDF1alpha/CXCR4 signaling stimulates beta-catenin transcriptional activity in rat neural progenitors. Neurosci. Lett. 398, 291–295, 10.1016/j.neulet.2006.01.024 (2006). 16469439

[b11] Tran P. B., Ren D., Veldhouse T. J. & Miller R. J. Chemokine receptors are expressed widely by embryonic and adult neural progenitor cells. J. Neurosci. Res. 76, 20–34, 10.1002/jnr.20001 (2004). 15048927

[b12] Takeuchi H. *et al.* Intravenously transplanted human neural stem cells migrate to the injured spinal cord in adult mice in an SDF-1- and HGF-dependent manner. Neurosci. Lett. 426, 69–74, 10.1016/j.neulet.2007.08.048 (2007). 17884290

[b13] Maucksch C. *et al.* IGF-I redirects doublecortin-positive cell migration in the normal adult rat brain. Neuroscience 241, 106–115, 10.1016/j.neuroscience.2013.03.021 (2013). 23528977

[b14] Xu Q. *et al.* Hypoxia-induced astrocytes promote the migration of neural progenitor cells via vascular endothelial factor, stem cell factor, stromal-derived factor-1alpha and monocyte chemoattractant protein-1 upregulation *in vitro*. Clin. Exp. Pharmacol. Physiol. 34, 624–631, 10.1111/j.1440-1681.2007.04619.x (2007). 17581219

[b15] Wang Y. *et al.* VEGF-overexpressing transgenic mice show enhanced post-ischemic neurogenesis and neuromigration. J. Neurosci. Res. 85, 740–747, 10.1002/jnr.21169 (2007). 17243175

[b16] Hirota Y. *et al.* Cyclin-dependent kinase 5 is required for control of neuroblast migration in the postnatal subventricular zone. J. Neurosci. 27, 12829–12838, 10.1523/JNEUROSCI.1014-07.2007 (2007). 18032654 PMC6673299

[b17] Francis F. *et al.* Doublecortin is a developmentally regulated, microtubule-associated protein expressed in migrating and differentiating neurons. Neuron 23, 247–256, S0896-6273(00)80777-1 (1999). 10399932 10.1016/s0896-6273(00)80777-1

[b18] Reiner O. *et al.* DCX’s phosphorylation by not just another kinase (JNK). Cell Cycle 3, 747–751 (2004). 15118415

[b19] Moors M., Cline J. E., Abel J. & Fritsche E. ERK-dependent and -independent pathways trigger human neural progenitor cell migration. Toxicol. Appl. Pharmacol. 221, 57–67, 10.1016/j.taap.2007.02.018 (2007). 17445854

[b20] Sonego M. *et al.* Fascin regulates the migration of subventricular zone-derived neuroblasts in the postnatal brain. J. Neurosci. 33, 12171–12185, 10.1523/JNEUROSCI.0653-13.2013 (2013). 23884926 PMC3721833

[b21] Huang Y. S. *et al.* Involvement of SHP2 in focal adhesion, migration and differentiation of neural stem cells. Brain Dev. 34, 674–684, 10.1016/j.braindev.2011.10.011 (2012). 22118986

[b22] Zhang C. *et al.* RhoC involved in the migration of neural stem/progenitor cells. Cell. Mol. Neurobiol. 34, 409–417, 10.1007/s10571-014-0026-0 (2014). 24414340 PMC11488953

[b23] Irving E. A. & Bamford M. Role of mitogen-and stress-activated kinases in ischemic injury. J. Cereb. Blood Flow Metab. 22, 631–647, 10.1097/00004647-200206000-00001 (2002). 12045661

[b24] Irving E. A., Barone F. C., Reith A. D., Hadingham S. J. & Parsons A. A. Differential activation of MAPK/ERK and p38/SAPK in neurones and glia following focal cerebral ischaemia in the rat. Brain Res. Mol. Brain Res. 77, 65–75 (2000). 10814833 10.1016/s0169-328x(00)00043-7

[b25] Walton K. M. *et al.* Activation of p38MAPK in microglia after ischemia. J. Neurochem. 70, 1764–1767 (1998). 9523596 10.1046/j.1471-4159.1998.70041764.x

[b26] Barone F. C. *et al.* Inhibition of p38 mitogen-activated protein kinase provides neuroprotection in cerebral focal ischemia. Med. Res. Rev. 21, 129–145 (2001). 11223862 10.1002/1098-1128(200103)21:2<129::aid-med1003>3.0.co;2-h

[b27] Kummer J. L., Rao P. K. & Heidenreich K. A. Apoptosis induced by withdrawal of trophic factors is mediated by p38 mitogen-activated protein kinase. J. Biol. Chem. 272, 20490–20494 (1997). 9252360 10.1074/jbc.272.33.20490

[b28] Sato K., Hamanoue M. & Takamatsu K. Inhibitors of p38 mitogen-activated protein kinase enhance proliferation of mouse neural stem cells. J. Neurosci. Res. 86, 2179–2189, 10.1002/jnr.21668 (2008). 18338804

[b29] Kim J. & Wong P. K. Loss of ATM impairs proliferation of neural stem cells through oxidative stress-mediated p38 MAPK signaling. Stem Cells 27, 1987–1998, 10.1002/stem.125 (2009). 19544430

[b30] Yang S. R. *et al.* NPC1 gene deficiency leads to lack of neural stem cell self-renewal and abnormal differentiation through activation of p38 mitogen-activated protein kinase signaling. Stem Cells 24, 292–298, 10.1634/stemcells.2005-0221 (2006). 16099992

[b31] Zhang D., Guo M., Zhang W. & Lu X. Y. Adiponectin stimulates proliferation of adult hippocampal neural stem/progenitor cells through activation of p38 mitogen-activated protein kinase (p38MAPK)/glycogen synthase kinase 3beta (GSK-3beta)/beta-catenin signaling cascade. J. Biol. Chem. 286, 44913–44920, 10.1074/jbc.M111.310052 (2011). 22039048 PMC3247954

[b32] Cheng A., Chan S. L., Milhavet O., Wang S. & Mattson M. P. p38 MAP kinase mediates nitric oxide-induced apoptosis of neural progenitor cells. J. Biol. Chem. 276, 43320–43327, 10.1074/jbc.M107698200 (2001). 11555660

[b33] Kendall S. E. *et al.* NRAGE mediates p38 activation and neural progenitor apoptosis via the bone morphogenetic protein signaling cascade. Mol. Cell. Biol. 25, 7711–7724, 10.1128/MCB.25.17.7711-7724.2005 (2005). 16107717 PMC1190310

[b34] Sheng W. S. *et al.* TNF-alpha-induced chemokine production and apoptosis in human neural precursor cells. Journal of leukocyte biology 78, 1233–1241, 10.1189/jlb.0405221 (2005). 16314440

[b35] Androutsellis-Theotokis A. *et al.* Notch signalling regulates stem cell numbers *in vitro* and *in vivo*. Nature 442, 823–826, 10.1038/nature04940 (2006). 16799564

[b36] Segarra J., Balenci L., Drenth T., Maina F. & Lamballe F. Combined signaling through ERK, PI3K/AKT, and RAC1/p38 is required for met-triggered cortical neuron migration. J. Biol. Chem. 281, 4771–4778, 10.1074/jbc.M508298200 (2006). 16361255

[b37] Behrens J., Kameritsch P., Wallner S., Pohl U. & Pogoda K. The carboxyl tail of Cx43 augments p38 mediated cell migration in a gap junction-independent manner. Eur. J. Cell Biol. 89, 828–838, 10.1016/j.ejcb.2010.06.003 (2010). 20727616

[b38] Zohn I. E. *et al.* p38 and a p38-interacting protein are critical for downregulation of E-cadherin during mouse gastrulation. Cell 125, 957–969, 10.1016/j.cell.2006.03.048: (2006). 16751104

[b39] Tamura Y. *et al.* Multi-directional differentiation of doublecortin- and NG2-immunopositive progenitor cells in the adult rat neocortex *in vivo*. Eur. J. Neurosci. 25, 3489–3498, 10.1111/j.1460-9568.2007.05617.x (2007). 17610569

[b40] Hamanoue M., Ikeda Y., Ogata T. & Takamatsu K. Predominant expression of N-acetylglucosaminyltransferase V (GnT-V) in neural stem/progenitor cells. Stem Cell Res 14, 68–78, 10.1016/j.scr.2014.11.004 (2015). 25524127

[b41] Hamanoue M. *et al.* Cell surface N-glycans mediated isolation of mouse neural stem cells. J. Neurochem. 110, 1575–1584, 10.1111/j.1471-4159.2009.06256.x (2009). 19573022

[b42] Schmidt N., Mishra A., Lai G. H. & Wong G. C. Arginine-rich cell-penetrating peptides. FEBS Lett. 584, 1806–1813, 10.1016/j.febslet.2009.11.046 (2010). 19925791

[b43] Zarubin T. & Han J. Activation and signaling of the p38 MAP kinase pathway. Cell Res. 15, 11–18, 10.1038/sj.cr.7290257 (2005). 15686620

[b44] Rizzuti M., Nizzardo M., Zanetta C., Ramirez A. & Corti S. Therapeutic applications of the cell-penetrating HIV-1 Tat peptide. Drug Discov Today 20, 76–85, 10.1016/j.drudis.2014.09.017 (2015). 25277319

[b45] Tran P. B. & Miller R. J. Chemokine receptors: signposts to brain development and disease. Nat. Rev. Neurosci. 4, 444–455, 10.1038/nrn1116 (2003). 12778117

[b46] Chen Y., Wei Y., Liu J. & Zhang H. Chemotactic responses of neural stem cells to SDF-1alpha correlate closely with their differentiation status. J. Mol. Neurosci. 54, 219–233, 10.1007/s12031-014-0279-6 (2014). 24659235

[b47] Lindvall O., Kokaia Z. & Martinez-Serrano A. Stem cell therapy for human neurodegenerative disorders-how to make it work. Nat. Med. 10 Suppl, S42–50, 10.1038/nm1064 (2004). 15272269

[b48] Naka-Kaneda H. *et al.* The miR-17/106-p38 axis is a key regulator of the neurogenic-to-gliogenic transition in developing neural stem/progenitor cells. Proc. Natl. Acad. Sci. USA 111, 1604–1609, 10.1073/pnas.1315567111 (2014). 24474786 PMC3910627

[b49] Hamanoue M., Sato K. & Takamatsu K. Inhibition of p38 mitogen-activated protein kinase-induced apoptosis in cultured mature oligodendrocytes using SB202190 and SB203580. Neurochem. Int. 51, 16–24, 10.1016/j.neuint.2007.03.005 (2007). 17459526

[b50] Mullen L. M. *et al.* Ras/p38 and PI3K/Akt but not Mek/Erk signaling mediate BDNF-induced neurite formation on neonatal cochlear spiral ganglion explants. Brain Res. 1430, 25–34, 10.1016/j.brainres.2011.10.054 (2012). 22119396 PMC3242932

[b51] Willaime S. *et al.* Ceramide-induced apoptosis in cortical neurons is mediated by an increase in p38 phosphorylation and not by the decrease in ERK phosphorylation. Eur. J. Neurosci. 13, 2037–2046 (2001). 11422444 10.1046/j.0953-816x.2001.01581.x

[b52] Noguchi Y. *et al.* Astrocytes protect neurons against methylmercury via ATP/P2Y(1) receptor-mediated pathways in astrocytes. Plos One 8, e57898, 10.1371/journal.pone.0057898 (2013). 23469098 PMC3585279

[b53] Lee Y. B., Schrader J. W. & Kim S. U. p38 map kinase regulates TNF-alpha production in human astrocytes and microglia by multiple mechanisms. Cytokine 12, 874–880, 10.1006/cyto.2000.0688 (2000). 10880231

[b54] Matsushita Y., Nakajima K., Tohyama Y., Kurihara T. & Kohsaka S. Activation of microglia by endotoxin suppresses the secretion of glial cell line-derived neurotrophic factor (GDNF) through the action of protein kinase C alpha (PKCalpha) and mitogen-activated protein kinases (MAPKS). J. Neurosci. Res. 86, 1959–1971, 10.1002/jnr.21657 (2008). 18438912

[b55] Miyake T. *et al.* TRPM2 contributes to LPS/IFNgamma-induced production of nitric oxide via the p38/JNK pathway in microglia. Biochem. Biophys. Res. Commun. 444, 212–217, 10.1016/j.bbrc.2014.01.022 (2014). 24462864

[b56] Kim K. Y. *et al.* Thrombin induces IL-10 production in microglia as a negative feedback regulator of TNF-alpha release. Neuroreport 13, 849–852 (2002). 11997699 10.1097/00001756-200205070-00022

[b57] Katayama T. *et al.* Accumulating microglia phagocytose injured neurons in hippocampal slice cultures: involvement of p38 MAP kinase. Plos One 7, e40813, 10.1371/journal.pone.0040813 (2012). 22815830 PMC3398896

[b58] Kobayashi K. *et al.* P2Y12 receptor upregulation in activated microglia is a gateway of p38 signaling and neuropathic pain. J. Neurosci. 28, 2892–2902, 10.1523/JNEUROSCI.5589-07.2008 (2008). 18337420 PMC6670682

[b59] Tsuda M., Mizokoshi A., Shigemoto-Mogami Y., Koizumi S. & Inoue K. Activation of p38 mitogen-activated protein kinase in spinal hyperactive microglia contributes to pain hypersensitivity following peripheral nerve injury. Glia 45, 89–95, 10.1002/glia.10308 (2004). 14648549

[b60] Ishiguro S. *et al.* Basic fibroblast growth factor induces down-regulation of alpha-smooth muscle actin and reduction of myofibroblast areas in open skin wounds. Wound Repair Regen. 17, 617–625, 10.1111/j.1524-475X.2009.00511.x (2009). 19614927

[b61] Nagahara H. *et al.* Transduction of full-length TAT fusion proteins into mammalian cells: TAT-p27Kip1 induces cell migration. Nat. Med. 4, 1449–1452, 10.1038/4042 (1998). 9846587

[b62] Nemeth E., Bole-Feysot C. & Tashima L. S. Suppression subtractive hybridization (SSH) identifies prolactin stimulation of p38 MAP kinase gene expression in Nb2 T lymphoma cells: molecular cloning of rat p38 MAP kinase. J. Mol. Endocrinol. 20, 151–156 (1998). 9513091 10.1677/jme.0.0200151

[b63] Han J., Lee J. D., Bibbs L. & Ulevitch R. J. A. MAP kinase targeted by endotoxin and hyperosmolarity in mammalian cells. Science 265, 808–811 (1994). 7914033 10.1126/science.7914033

[b64] Webb S. E., Pollard J. W. & Jones G. E. Direct observation and quantification of macrophage chemoattraction to the growth factor CSF-1. J. Cell Sci. 109 (Pt 4), 793–803 (1996). 8718671 10.1242/jcs.109.4.793

