# Supplementary material for: Cell-permeable p38 MAP kinase promotes migration of adult neural stem/progenitor cells
Source: Sci Rep. 2016 Apr 12;6:24279. doi: 10.1038/srep24279 (PMC4828673; doi:10.1038/srep24279)
Supplement: Supplementary Information [file srep24279-s1.pdf]

Supplemental Information

Title

Cell-permeable p38 MAP kinase promotes migration of adult neural stem/progenitor cells

Makoto Hamanoue[1,2]*, Kazuhito Morioka[3], Ikuroh Ohsawa[4], Keiko Ohsawa[5], Masaaki Kobayashi[1], Kayo Tsuburaya[6], Yoshikiyo Akasaka[2,6], Tetsuo Mikami[6], Toru Ogata[7], and Ken Takamatsu[1,2]

Supplemental Fig. 1. Adult cultured NPCs express activated p38 protein. Immunocytochemical analysis revealed that both nestin-positive (a, arrows) and Dcx-positive NPCs (b, arrows) expressed phosphorylated p38. Scale bar = 20 μm.

Supplemental Fig. 2. Cell-permeable p38 protein did not influence survival of adult NPCs. Adult NPCs were cultured for 16 hr (a, n = 4–10) or 5 days (b, n = 9) in growth-promoting medium containing cell-permeable p38 proteins at the indicated concentrations (a) or at 300 nM (b). The number of cells following treatment with

PTD-p38WT is expressed relative to the number in the control sample. Data are means ± SEM. Cell-permeable p38 proteins did not influence cell number.

Supplemental Fig. 3. Cell-permeable p38 protein did not influence differentiation of adult NPCs. Adult NPCs were cultured for 5 days in growth-promoting medium containing cell-permeable p38 proteins (300 nM). Western blot analysis of cell lysates was performed using antibodies against cell type–specific antigens (n = 3–4); BLBP for NPCs (a, b), Dcx for migrating NPCs (c, d), NeuN for neurons (e, f), and GFAP for astrocytes (g, h). Protein levels were normalized against actin (i). No significant changes in expression levels of cell-specific antigens were observed between cells treated with PTD-p38WT and control cells (a–h). Only PTD-p38KD inhibited GFAP expression (g, h). **$P < 0.01$.

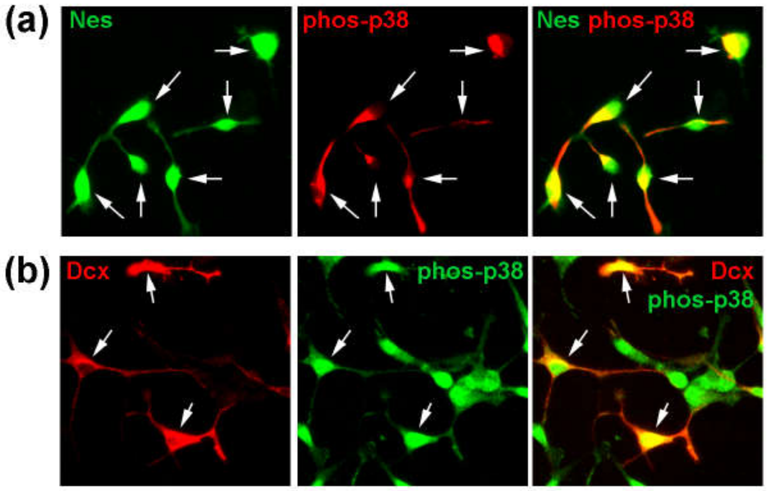
(a)
Nes
phos-p38
Nes phos-p38
(b)
Dcx
phos-p38
Dcx
phos-p38

(a) 16h

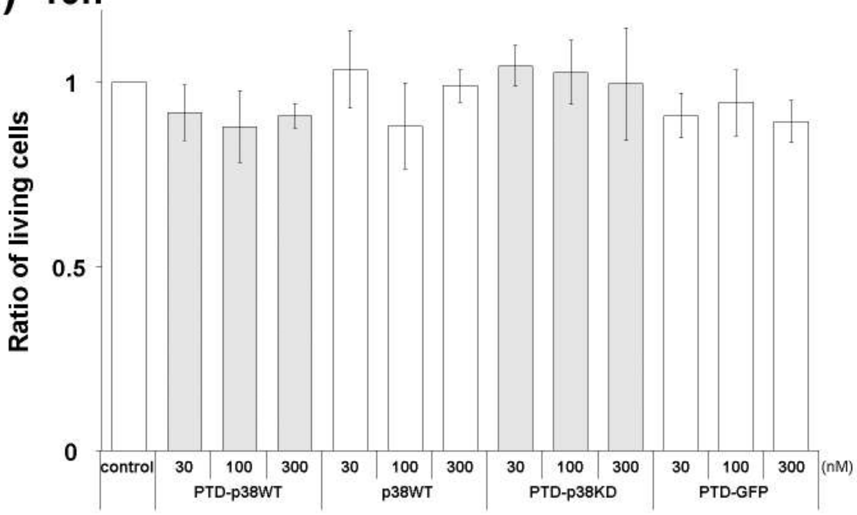

Ratio of living cells
1
0.5
0
control
30
100
300
30
100
300
30
100
300
30
100
300
(nM)
PTD-p38WT
p38WT
PTD-p38KD
PTD-GFP

(b) 5 days

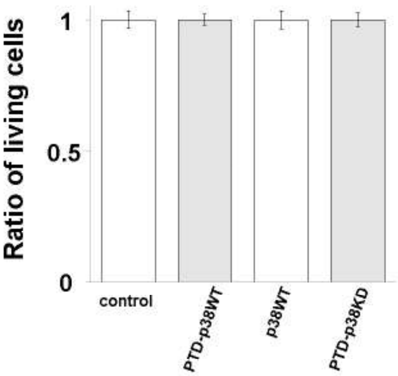

Ratio of living cells
1
0.5
0
control
PTD-p38WT
p38WT
PTD-p38KD

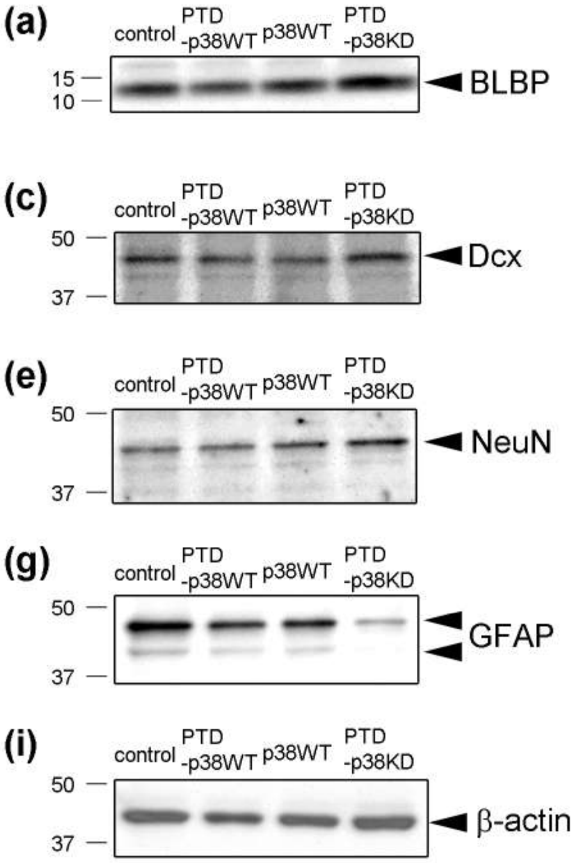

(a)
control
PTD -p38WT
p38WT
PTD -p38KD
15
10
BLBP
(b)
Relative intensity
1.0
0.5
0.0
control
PTD -p38WT
p38WT
PTD -p38KD
(c)
control
PTD -p38WT
p38WT
PTD -p38KD
50
37
Dcx
(d)
Relative intensity
1.0
0.5
0.0
control
PTD -p38WT
p38WT
PTD -p38KD
(e)
control
PTD -p38WT
p38WT
PTD -p38KD
50
37
NeuN
(f)
Relative intensity
1.5
1.0
0.5
0.0
control
PTD -p38WT
p38WT
PTD -p38KD
(g)
control
PTD -p38WT
p38WT
PTD -p38KD
50
37
GFAP
(h)
Relative intensity
1.0
0.5
0.0
**
control
PTD -p38WT
p38WT
PTD -p38KD
(i)
control
PTD -p38WT
p38WT
PTD -p38KD
50
37
β-actin
